# A specific inhibitor of protein kinase CK2 delays gamma-H2Ax foci removal and reduces clonogenic survival of irradiated mammalian cells

**DOI:** 10.1186/1748-717X-6-15

**Published:** 2011-02-10

**Authors:** Felix Zwicker, Maren Ebert, Peter E Huber, Jürgen Debus, Klaus-Josef Weber

**Affiliations:** 1 Department of Radiation Oncology, University of Heidelberg, Heidelberg, Germany; 2 Clinical Cooperation Unit Radiation Oncology, DKFZ, Heidelberg, Germany

## Abstract

**Background:**

The protein kinase CK2 sustains multiple pro-survival functions in cellular DNA damage response and its level is tightly regulated in normal cells but elevated in cancers. Because CK2 is thus considered as potential therapeutic target, DNA double-strand break (DSB) formation and rejoining, apoptosis induction and clonogenic survival was assessed in irradiated mammalian cells upon chemical inhibition of CK2.

**Methods:**

MRC5 human fibroblasts and WIDR human colon carcinoma cells were incubated with highly specific CK2 inhibitor 4,5,6,7-tetrabromobenzotriazole (TBB), or mock-treated, 2 hours prior to irradiation. DSB was measured by pulsed-field electrophoresis (PFGE) as well as gamma-H2AX foci formation and removal. Apoptosis induction was tested by DAPI staining and sub-G1 flow cytometry, survival was quantified by clonogenic assay.

**Results:**

TBB treatment did not affect initial DNA fragmention (PFGE; up to 80 Gy) or foci formation (1 Gy). While DNA fragment rejoining (PFGE) was not inhibited by the drug, TBB clearly delayed gamma-H2AX foci disappearence during postirradiation incubation. No apoptosis induction could be detected for up to 38 hours for both cell lines and exposure conditions (monotherapies or combination), but TBB treatment at this moderately toxic concentration of 20 μM (about 40% survival) enhanced radiation-induced cell killing in the clonogenic assay.

**Conclusions:**

The data imply a role of CK2 in gamma-H2AX dephosporylation, most likely through its known ability to stimulate PP2A phosphatase, rather than DSB rejoining. The slight but definite clonogenic radiosensitization by TBB does apparently not result from interference with an apoptosis suppression function of CK2 in these cells but could reflect inhibitor-induced uncoupling of DNA damage response decay from break ligation.

## Introduction

Protein kinase CK2 is a ubiquitous and highly conserved protein serine/threonine kinase with a broad spectrum of target proteins the majority of which play a role in signal transduction and gene expression promoting cell survival upon phosphorylation [1-3]. CK2 predominantly exists as heteroterameric holoenzyme and, in mammalian cells, basal activity of CK2 is conferred by intra-molecular interaction of either catalytic isoform CK2α or CK2α' and may be regulated via the association with a dimer of the regulatory CK2β subunit (ααβ_2_, α'α'β_2_, or αα'β_2_) [2,4]. CK2 is dysregulated in most cancers that have been examined with high levels found particularly in the nuclear compartment [2,5,6]. While the precise roles of CK2 in tumorigenesis are still not completely understood, anti-apoptosis functions of CK2 through the regulation of tumor suppressor and oncogene activity have been suggested [6], and CK2 is now considered as a potential therapeutic target [7].

Ionizing radiation-induced DNA double-strand breaks provoke a complex cellular response which activates and coordinates cell-cycle checkpoints, damage repair, and the eventual onset of apoptosis [8]. The DNA damage response (DDR) invokes chromatin structure changes extending over megabasepair regions flanking a DSB, particularly phosphorylations of the histone variant H2AX [9], as well as the concomitant accumulation of the diverse factors mediating DNA damage signaling [10]. Their visualization by means of immunostaining and fluorescence microscopy (the socalled "focus assay") has thus become the prevailing method of tracking DSB and dissecting the activating and regulatory components of this signal amplification. CK2 targets several components of the DDR by constitutive as well as damage induced phosphorylations:

(i) within the major nonhomologous end joining (NHEJ) pathway of DSB repair [11], the Xrcc4 protein, an adaptor for DNA ligase IV, was shown to recruit DNA end-processing factors requiring CK2-dependent constitutive phosphorylation [12]. This implied a role for CK2 in the DSB rejoining reaction reminiscent of CK2-dependent activation of Xrcc1 in single-strand break repair [13].

(ii) another adaptor protein is MDC1 which, when phosphorylated by CK2, promotes assembly and retention of the MRE11-Rad50-Nijmegen breakage syndrome 1 (NBS1) [MRN] complex around sites of DSB, and in conjunction with DSB-induced ATM (ataxia telangiectasia mutated) kinase allows spreading of γH2AX formation [14-18].

(iii) a new DSB-induced function of CK2 in chromatin modification was recently described due to heterochromatin protein 1 (HP1-β) phosphorylation (19). HP1 is a critical factor for chromatin compaction being recruited by direct interaction with H3K9me (trimethylated lysine 9 of histone H3), an epigenetic mark for silenced chromatin (20). It was shown that CK2-phosphorylated HP1-β looses its affinity for H3K9me suggesting a relieve of the structural constraints within compacted chromatin that prevent the access of DDR factors [19,21]. Accordingly, CK2 inhibition by TBB suppressed HP1-β mobilization and diminished ATM-dependent H2AX phosphorylation.

Less data is available on the phenotypic expression of such CK2-dependent interactions. An increased apoptotic response was reported for irradiated HeLa cells when subjected to siRNA-mediated CK2 depletion, without affecting the radiation-induced G2-M checkpoint [22]. Other authors found an increased radiation sensitivity due to a mutation of the CK2 consensus site in Xrcc4 both for clonogenic survival and γH2AX focus removal but did not assess fragment rejoining [12]. With the current study we thought to gain additional data on DSB repair, measured with both the PFGE and the γH2AX focus assay, apoptosis induction and clonogenic survival after ionizing radiation exposure in response to treatment with the highly specific CK2 inhibitor 4,5,6,7-tetrabromobenzotriazole (TBB) [23]. Experiments were conducted with human fibroblasts and a human colon carcinoma cell line.

## Materials and methods

### Cell lines and culture conditions

MRC5 (human lung fibroblasts; BioWhittacker, Verviers, Belgium) and WIDR cells (human colon carcinoma; Tumorbank of the German Cancer Research Center, Heidelberg) were maintained in RPMI 1640 (MRC5) or DMEM (WIDR) with 10% fetal calf serum (Biochrom, Berlin, Germany) containing 1% L-glutamine (Serva, Heidelberg, Germany). Cells were grown as monolayers in humidified 6% CO_2_/air at 37°C. Under these conditions cell cultures exhibited doubling times of 48 hours (MRC5) or 28 hours (WIDR), respectively. The plating efficiencies ranged from 70-90% for the WIDR tumor cells and from 1-2.5% for the MRC5 fibroblasts.

### Drug treatment and irradiation

Aliquots of 10 mM stock solution of the CK2 inhibitor TBB (Calbiochem, Merck Darmstadt, Germany) in DMSO were were stored at -20°C. For combined exposures, TBB was added to cultures at a desired concentration 2 hours prior to irradiation (also unirradiated controls). Mock treatments were performed by adjusting the respective DMSO concentration to that of the TBB samples. Irrradiations of cell cultures were conducted at a clinical linear accelerator (6 MV photon mode, 2.5 Gy/min).

### Pulsed-field electrophoresis

Measurement of DSB induction and rejoining was done by pulses-field-gel-electrophoresis (PFGE) as described, earlier [24]. Late log-phase cells (>80% confluency) were treated for 2 hours with 20 μM TBB (0,2% DMSO) or only DMSO before irradiation on ice and sample preparation. Alternatively, cells were incubated for repair prior to lysis. Data analysis involved quantification of the fraction of total DNA mass in electrophoretically mobile DNA fragments (FR-values) by means of ethidium bromide DNA staining and a digital gel image acquisition and analysis system (UVP Ltd., Cambridge, UK). FR-values obtained after incubation for repair were transformed into the respective radiation doses at which such values had been measured without repair ("dose-equivalents").

### Immunofluorescence

Antibodies were mouse anti-γH2AX^Ser139 ^(Upstate, Buckingham, U.K.), mouse anti-CK2 (á-subunit) (Calbiochem, Merck KGaA, Darmstadt, Germany) and Alexa Fluor R 488 goat anti-mouse secondary antibody (Molecular Probes, Eugene, Oregon, USA). Cells grown on coverslips were fixed (3% paraformaldehyde, 2% sucrose/PBS for 10 min at room temperature) and permeabilized (20 mM HEPES (pH 7.4), 50 mM NaCl, 3 mM MgCl_2_, 300 mM sucrose, and 0.5% Triton X-100 for 5 min at 4°C; chemicals from Sigma-Aldrich, Taufkirchen, Germany). Coverslips were washed in PBS before immunostaining. Primary antibody incubations with anti-γH2AX or with anti-CK2α' were performed for 40 min at 37°C at 1:500 dilution (2 μg/ml)) in PBS supplemented with 2% bovine serum fraction V albumin (Sigma-Aldrich) and followed by washing 4 times in PBS. Incubations with fluorochrome-labelled secondary antimouse antibodies were performed at 37°C at 1:200 dilution (10 μg/ml) in 2% bovine serum fraction V albumin for 20 min. Nuclei were counterstained in Vectashield mounting medium with DAPI (Vector Laboratories, Peterborough, United Kingdom) for 5 min at 20°C. Formation and time-dependent disappearence of distinct γH2AX-foci was scored in cells irradiated with 1 Gy (+/- 20 μM TBB pretreatment) by means of fluorescence microscopy (100× magnification and manual focal plane scanning). Foci present in 50 cells were counted for each sample.

### Flow cytometry and apoptosis measurement

Treated cells (including a TBB treatment extended to 8 hours before irradiation) were harvested at different times including the culture supernatant and were prepared for DNA flow cytometry (FacScan/Cell-Quest Pro, Beckton-Dickinson, Heidelberg, Germany) by propidium-iodide staining, aimed at either the measurement of cell cycle distribution or sub-G1 analysis, according to a standardized protocol [25]. Additionally, cells grown on microscopic slides were stained with DAPI (4,6-diamidino-2-phenylindol) at different post-treatment periods, and the nuclei were inspected for the typical morphological appearance of chromatin condensation during late apoptosis [26].

TBB-treated cells were also inspected for the phosphorylation status of Xrcc1 at residues S518/T519/T523, known to be targeted by CK2 kinase [27,28], by means of flow cytometry after intracellular immunostaining. The respective phospho-Xrcc1 antibody (polyclonal rabbit anti-human IgG) was from Bethyl, Inc. (Biomol, Berlin Germany), secondary FITC-labelled goat anti-rabbit (Ig) was from BD Pharmingen (Heidelberg, Germany). Rabbit IgG isotype control (Imgenex) was obtained from Biomol. Immunostaining was performed according to the manufactures (Bethyl) instruction.

### Clonogenic assay

Clonogenicity of MRC5 and WIDR cells were measured by a standardized colony forming assay. For combined exposures, a fixed TBB concentration of 20 μM (0,2% DMSO) was used. This concentration of the solute resulted in only a small decrease of plating efficiencies (90% to 95% relative to the plating efficiency without DMSO for both cell lines), but samples without drug were always mock treated. Cells were plated in triplicate within a single experiment and at least three independent experiments were performed for each condition tested.

## Results

**Figure 1:**
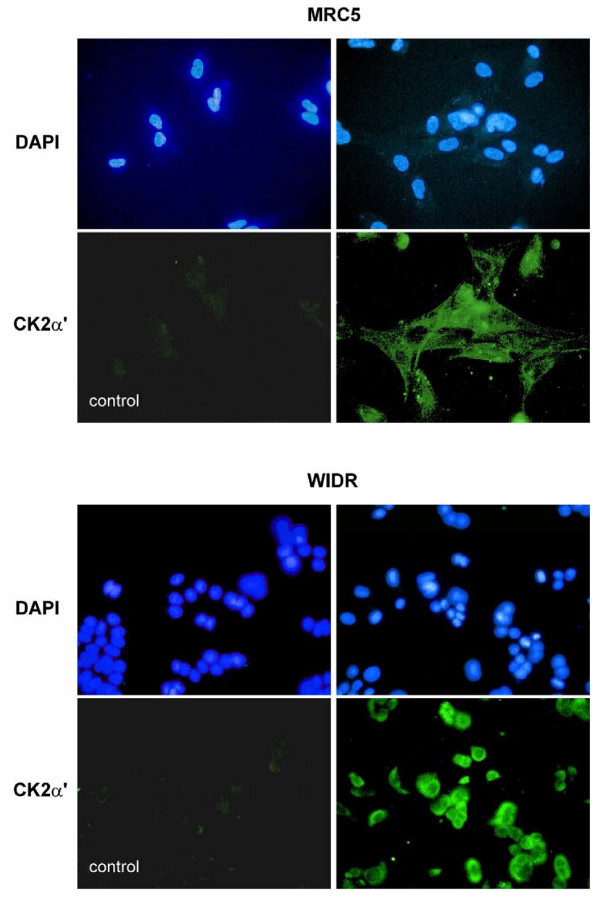
**Intracellular distribution of CK2 in MRC5 and WIDR cells as visualized by immunostaining with CK2α' subunit antibody (lower right panels)**. Lower left panels are isotype controls and the upper panels represent the respective DAPI counterstains (as indicated).

**Figure 2:**
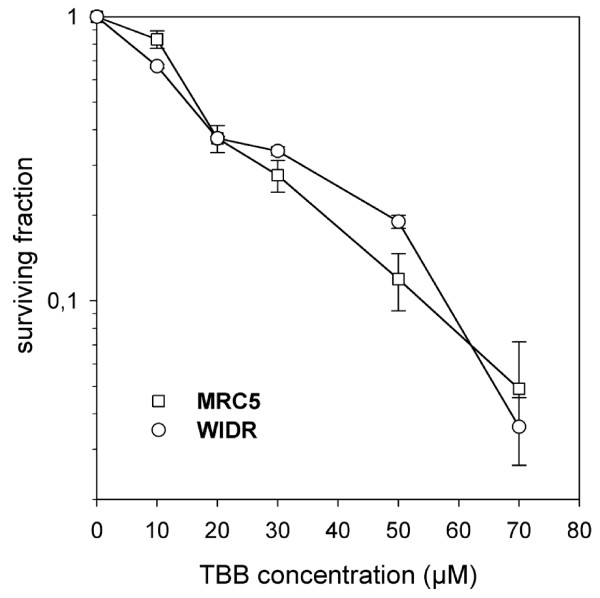
**Inhibition of clonogenic survival of MRC 5 and WIDR cells by a 2 hours incubation with the CK2 inhibitor 4,5,6,7-tetrabromobenzotriazole (TBB)**. Concentration of the TBB solute DMSO was adjusted to 0.7% (such as with the 70 μM TBB) for all samples. Data points represent mean values (and standard deviations) from three independent determinations.

**Figure 3:**
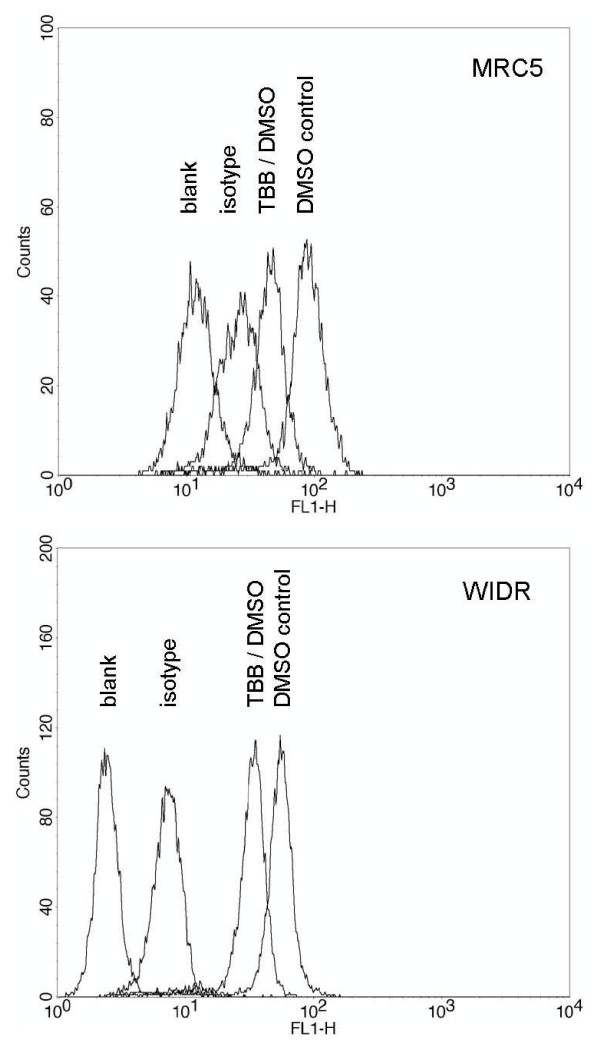
**Immunocytochemical measurement by flow cytometry of Xrcc1 phosphorylation status**. Cells were treated with 20 μM TBB (12 hours) and inspected for phospho-Xrcc1 staining (see Methods). Unstained samples are denoted as "blank". Samples prepared from MRC5 cells (upper panel) were measured at an increased amplifier gain to allow for a better representation of the difference between the respective histograms.

CK2 was expressed in MRC and in WIDR cells as detected by immunocytochemistry using CK2α' antibody and exhibited a strong preference to localize in the perinuclear compartment in the tumor cells (Figure 1). The effect of the CK2 inhibitor alone on clonogenic survival of the MRC5 and the WIDR cells is summarized in Figure 2. A common TBB exposure of 20 μM exhibiting definite but still moderate toxicity was chosen for the combination experiments. To test the inhibition of CK2 by this TBB concentration with a functional *in vivo *assay, the phosphorylation status of the Xrcc1 protein at 12 hours after addition of the drug was assessed as described, above. FACS histograms representing phospho-Xrcc1 staining with or without TBB treatment are shown in Figure 3 for MRC5 and WIDR cells. A distinct reduction of Xrcc1 phosphorylation due to TBB is evident.

### Pulsed field electrophoresis assay

**Figure 4:**
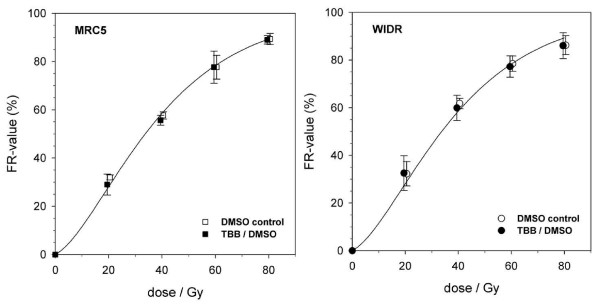
**Pulsed-field electrophoresis (PFGE) measurement of ionizing radiation-induced DNA double-strand breakage in MRC5 and WIDR cells (as indicated in the graphs)**. Fractions of electrophoretically mobile DNA (fragments < 9 Mbp), or FR-value, increases with dose according to the random breakage formalism (solid lines). DSB induction is not affected by TBB pretretment (200 μM for two hours) compared to the respective 2% DMSO controls. Data points represent mean values (and standard deviations) from three independent experiments.

**Figure 5:**
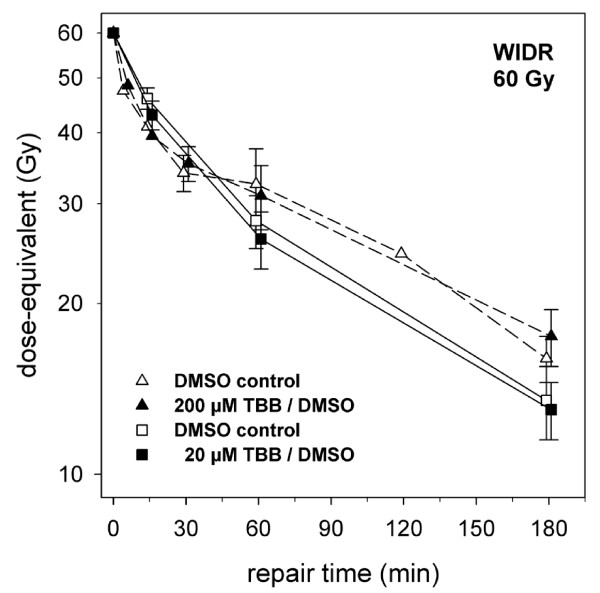
**Residual DNA fragmentation (represented as dose-equivalent values, see Methods section) after different periods of incubation for repair of WIDR cells in the presence of 20 μM TBB or 200 μM TBB (as indicated) added for 2 hours prior to irradiation with 60 Gy**. Data points represent mean values (and standard deviations) from three independent determinations.

Initial radiation-induced DSB yield (20 to 80 Gy) did not depend on 20 μM TBB pretreatment. Even for the high exposure conditions of 200 μM versus the DMSO control treatment, the respective dose-dependencies (+/- TBB) for the two cell lines were identical (Figure 4) with induction rates (from fits to the random breakage model [24,29]) of 0,0051 (MRC5) and 0,0052 (WIDR) DSB/Mbp/Gy, respectively. DSB rejoining during incubation for repair of up to 3 hours after an inducing dose of 60 Gy (represented as dose-equivalent values, as outlined in the Methods section) was also not affected by 20 μM TBB pretreatment of both WIDR and MRC5 cell lines. Suspecting that the low inhibitor concentration might have been insufficient to resolve a TBB-induced repair defect, measurements were also done at high exposure conditions (200 μM) within a separate set of experiments, but rejoining was again not inhibited. Because of the qualitative identy of the results obtained with the two cell lines, they are exemplified for the WIDR cells (Figure 5), only.

### γH2AX focus assay

**Figure 6:**
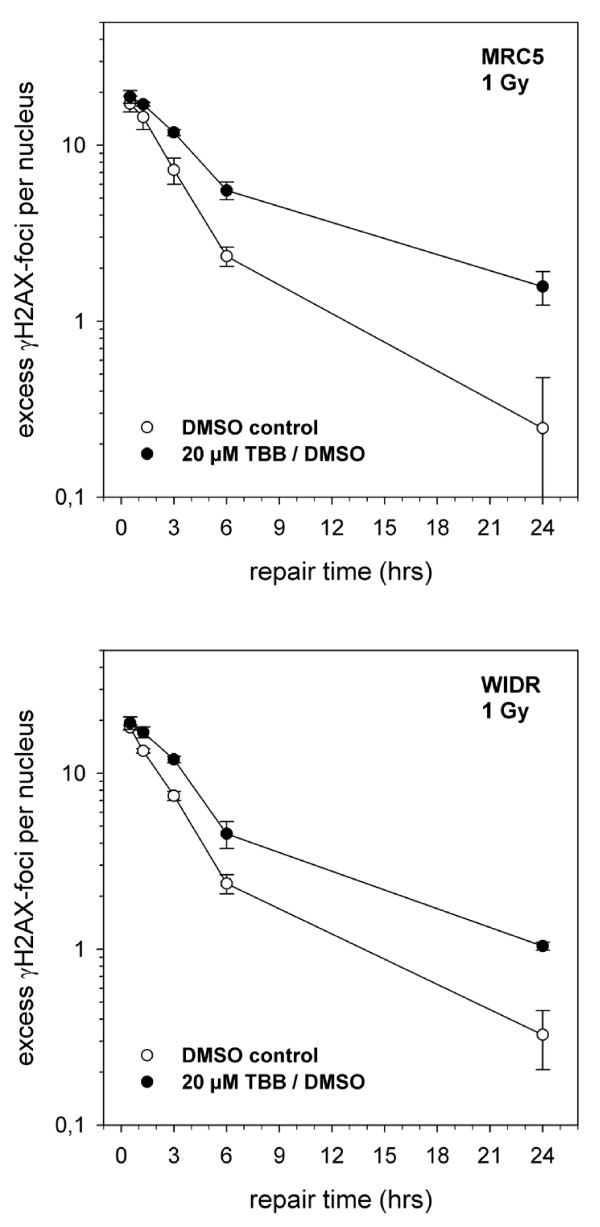
**Average number of γH2AX foci in the nuclei of MRC5 cells (upper panel) or WIDR cells (lower panel) after different periods of incubation for repair and in the presence of 20 μM TBB added for 2 hours prior to irradiation with 1 Gy**. Background numbers of foci were subtracted within each individual experiment (50 nuclei per treatment condition) before calculating the displayed mean values (and standard deviations) from at least 4 independent determinations.

TBB-dependent (20 μM) postirradiation foci disappearence (after 1 Gy) is depicted in Figure 6 for both the MRC5 and the WIDR cells. The number of background foci (unirradiated cells) was determined within each independent experiment and subtracted from the respective number of an irradiated sample. Notably, the average number of background foci per nucleus was not different in TBB/DMSO versus DMSO control samples (MRC5: 0.41 ± 0,15 versus 0.46 ± 0.14; WIDR: 0.6 ± 0.3 versus 0.54 ± 0.28). The data clearly demonstrates that TBB treatment did not reduce the number of initial foci after 1 Gy although the appearence of the individual foci was less bright (approximately reduced to 50-70%). Subsequent foci removal, however, was markedly delayed which is at variance with the results from the PFGE assay.

### Cell vitality

**Figure 7:**
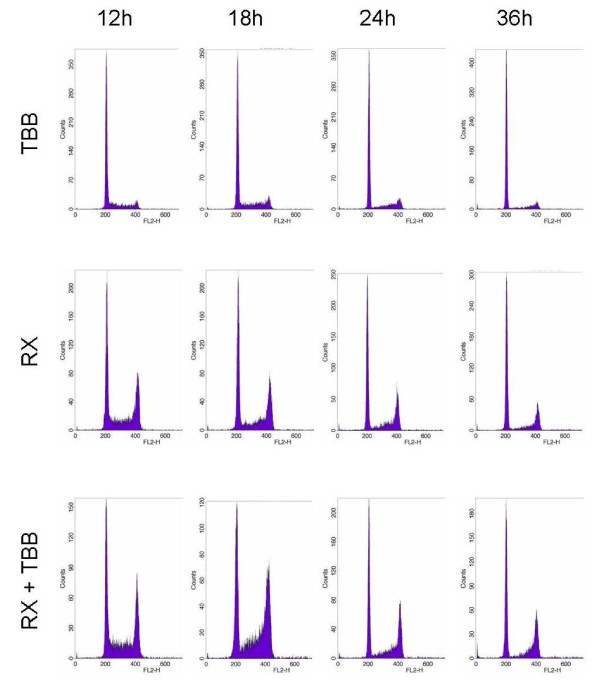
**Cell cycle measurements (FACS) at different times after 5 Gy irradiation of WIDR cells pretreated (2 hours) with 20 μM TBB (lower panels) or DMSO, only (middle panels)**. The upper panels show histograms after TBB treatment without irradiation which were identical to the respective DMSO controls (not shown).

**Figure 8:**
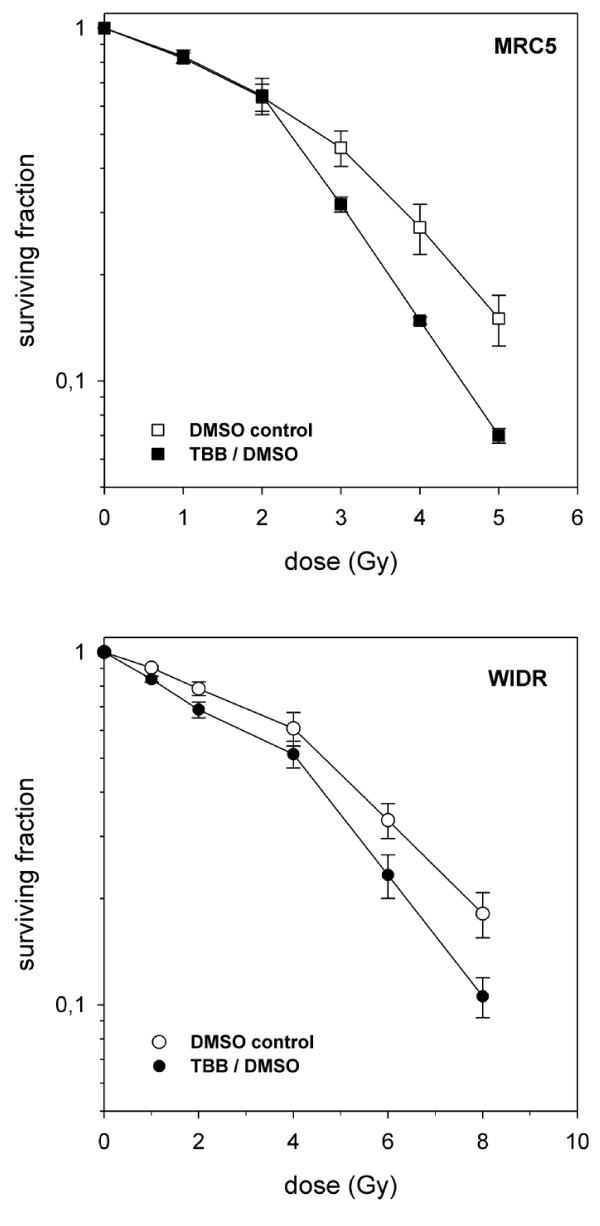
**Radiation-induced inhibition of clonogenic survival of MRC5 cells (upper panel) or WIDR cells (lower panel) in the presence of 20 μM TBB or only DMSO (as indicated) added for 2 hours prior to irradiation**. Data points represent mean values (and standard deviations) from 3 (MRC5) or 5 (WIDR) independent determinations.

Sub-G1 flow-cytometry or DAPI-staining of MRC5 or WIDR cells failed to demonstrate apoptosis induction over a period of up to 38 hours following 8 Gy irradiation, incubation with 40 μM TBB, or a combined exposure even when the TBB pretreatment period was extended to 8 hours (data not shown). Cell cycle analysis by flow cytometry was able to detect the well known radiation-induced G2-arrest (WIDR cells after 5 Gy: Figure 7) which was clearly more expressed and prolonged upon 20 μM TBB pretreatment. Radiation inhibition of clonogenic survival was enhanced by the CK2 inhibition with 20 μM TBB compared to 0.2% DMSO controls (normalized data in Figure 8). For the MRC5 cells, this effect was only observed with radiation doses ≥3 Gy.

## Discussion

CK2 (alpha') was expressed in both cell lines with a marked peri-nuclear accumulation in the tumor cell line. This finding is in accordance with the previously reported higher ratio of nuclear to cytosolic activity of CK2 in cancer cells than in normal cells [30]. Despite the observed difference in basal CK2 localization, the cytotoxic effect of CK2 inhibition was very similar with both cell types indicating that the subcellular distribution of CK2 was not the prevailing determinant of its pro-survival function. But this particular aspect was not further investigated.

A critical issue in the use of a chemical kinase inhibitor is its specificity for a given target enzyme. TBB was previously shown to exhibit a remarkable *in vitro *selectivity for CK2 among a panel of about 80 kinases, where only three other groups of kinases were inhibited by TBB with comparable efficacy [23]. They are not known to be involved in DSB processing but two of the respective kinases (HIPK2 and DYRK2) regulate p53-dependent differential transactivation of growth arrest genes versus pro-apoptotic genes in response to DNA damage severity [31]. The reported *in vitro *IC_50 _for TBB is 0.15 μM [23] which is a factor of about 100 lower than respective numbers obtained when cells were exposed to TBB before protein extraction and assessment of *in vitro *phosphorylation with synthetic CK2 target peptide [32,33]. The 2 hours exposure of cells at 20 μM TBB prior to irradiation used in the present study is therefore considered as an effective treatment for the reduction of CK2 activity while still being only moderately toxic in the clonogenic assay. The efficacy of this TBB exposure with respect to the inhibition of CK2 was further confirmed by a functional assay (Figure 3) that measured intracellular phosphorylation of a protein (Xrcc1) for which CK2 is known to be the major kinase [27,28].

The major result of the present investigation, however, is the discrepancy between DSB rejoining (PFGE) and the disappearence of γH2AX foci, where only the latter was delayed upon CK2 inhibition. A comparison of the results obtained with these two methods needs to consider the widely different doses applied. Analysis of DNA fragmentation requires sufficiently small fragments that may be resolved during electrophoresis and which are only produced at high doses. On the contrary, the focus assay is applicable at doses of only a few Gy. A comparison of absolute repair/rejoining rates derived with these different assay may thus be problematic. But particularly with the PFGE assay, it is not known that a repair modification, by whatever reason, would become differentially detectable depending on the dose level at which it is investigated.

Therefore, CK2-phosphorylated factors known to be involved in the response to radiation-induced DSB (Xrcc4, MDC1 or HP1-β; referred to in the Introduction section) need to be discussed.

Xrcc4 is constitutively phosphorylated at Thr233 by CK2 and this was shown to mediate the recruitment of DSB end-processing factors [12] which aid NHEJ [11]. Utilizing a Thr233 mutant system, it was also shown that lack of the CK2-targeted site resulted in a delayed removal of γH2Ax foci, but an actual rejoining defect, which may well have existed, was not assessed by these authors [12]. One has to keep in mind that the absence of constitutive CK2-dependent Xrcc4 phosphorylation represents a quite different situation compared to the addition of the CK2 inhibitor shortly (2 hours) prior to irradiation and assessment of DSB rejoining. Therefore, the phosphorylation status of Xrcc4 (at the CK2 residue) is sufficiently long-lived, or does not impact on NHEJ efficiency to an extent that could be resolved by our PFGE assay.

MDC1 is constitutively phosphorylated at multiple residues by CK2 [15]. But unlike Xrcc4, MDC1 functions - via MRN-complex recruitment - in the propagation and retention of the chromatin changes that spread over large regions surrounding a DSB, particularly the ATM-dependent H2AX phosphorylation (see: Introduction section), rather than being involved in the DSB rejoining reaction [11,15]. The present experiments could not detect an inhibitory effect of TBB on the number of initially formed γH2AX foci. This is in accordance with an earlier observation where TBB was unable to abrogate MRN recruitment (NBS1 foci) whereas downregulation of CK2 by siRNA was effective, leading to the conclusion that chemical CK2 inhibition was not potent enough to sufficiently reduce CK2 activity towards MDC1 [15].

Recently, ATM was shown to act as a repair factor for DSB in heterochromatic regions of the genome by phosphorylating heterochromatin protein KAP-1 to allow for localized and transient changes of chromatin organization that would otherwise inhibit repair [34-36]. Notably, knockdown of another heterochromatin protein, HP1-β, relieved the requirement for ATM in heterochromatic DSB repair [34,35]. HP1-β is phosphorylated by CK2 in response to DNA damage leading to its mobilization from chromatin and allowance for H2AX phosphorylation [19]. Accordingly, CK2 inhibition by 20 μM TBB resulted in a decreased fluorescence intensity of the individual γH2AX foci formed shortly after irradiation, which is at least qualitatively confirmed by our observation. Whether a TBB treatment affected DSB rejoining was not measured by these authors [19], but our results strongly argue against this possibility. Because foci did in fact form with initial numbers being independent from TBB preexposure, we conclude that the role of CK2 in the DNA damage response is to aid the propagation of chromatin changes, i.e. by the suggested mobilization of HP1, distal to the DSB site rather than being involved in initial damage recognition and rejoing. This appears to be different to the role of ATM in heterochromatin repair, where cells with defective or downregulated ATM fail to rejoin that particular portion (about 15%) of all induced DSB [37].

These considerations, however, do not answer the question why the disappearance of foci was delayed due to CK2 inhibition. γH2AX focus decay may proceed through i*n-situ *dephosphorylation or by histone exchange (followed by dephosphorylation of displaced γH2AX). In mammalian cells, protein phosphatase PP2A seems to be crucially involved in γH2AX dephosphorylation [38]. It was shown that PP2A directly interacts with CK2 catalytic subunit α kinase thus getting phosporylated and activated [39] implicating a role of CK2 in γH2AX turnover. The respective scheduling, however, is still controversial. The early decrease in the number of foci was not associated with a significant change in the global γH2AX level as measured by flow cytometry or western blotting [40] favouring the idea of a histone exchange mechanism being responsible for the dissolution of foci which would then not *a priori *require phosphatase activity. A similar conclusion was reached when the inhibition of PP2A by calyculin A, an agent known to suppress γH2AX dephosporylation [41], had only a small effect on foci elimination [42]. In contrast, other authors did in fact observe a strong inhibition of foci decay by PP2A inhibitor calyculin A following radiation exposure (10, 42). Notably, this finding was not accompanied by a respective rejoining defect (using PFGE analysis) similar to what is found in the present investigation. A slower removal of γH2AX foci was also noted when a more specific inhibitor of PP2A (fostriecin) was used, or when employing PP2A catalytic subunit knockdown [38]. In the latter study, foci formation was induced by stalling replication forks due to treatment with topoisomerase inhibitor camptothecin and, contrary to the radiation studies [10,43], a concurrently expressed repair defect was measured by means of neutral comet assay. Whether this discrepancy could relate to the different mechanisms of damage induction or the distinct experimental approaches to assess residual breakage remains unclear.

The present data together with the above considerations argue for an involvement of CK2 in DNA damage response relaxation through its ability to stimulate *in-situ *γH2AX dephosphorylation, most likely via PP2A recruitment/activation. Because foci decay is thought to reflect a timely response to finalized damage repair, CK2 appears to exert a respective coordinating function which can be uncoupled upon its chemical inhibition whereby the end joining reaction is not affected (at least under the experimental conditions, used here). The apparent increase and persistance of the DNA damage checkpoint function at the G2/M border when CK2 was inhibited (as demonstrated for the WIDR cells in Figure 7) would be consistent with this idea.

The slight, though definitive, increase of clonogenic radiosensitivity of both cell lines when CK2 was inhibited could thus relate to an imbalanced DNA damage response. Different from an earlier study using HeLa tumor cells with CK2 being depleted by means of RNA interference [22], this effect was not due to triggering apoptosis which could potentially result from persistant damage signaling. Accordingly, the present observations reflect the modification of other major mechanisms through which clonogenicity upon irradiation is abrogated, i.e. a permanent growth arrest such as expressed in non-transformed fibroblasts or the prevailing mitotic catastrophe when cells bearing residual DNA damage enter mitosis [44]. While a persistant damage signaling could in fact enhance permanent growth arrest (such as in the fibroblasts), it is not immediately clear how a prolongation of the radiation-induced G2/M transition delay (at undisturbed repair efficacy) would potentiate mitotic failures. CK2 has been implicated in the efficacy of either checkpoint by interactions with other key regulatory elements including the tumor suppressor p53 [45,46] or the phosphatase cdc25B and C isoforms [47,48]. But given the highly promiscious nature of CK2, other coordinating factors of cell cycle progression after DNA damage (i.e. SMC3 for intra-S phase checkpoint [49]) may have been affected by the TBB treatment, as well. The phenotypes of increased radiation sensitivities of the two cell systems may thus reflect distinct and differentially CK2-dependent factors of the DNA damage response. This could tentatively explain the intriguing qualitative difference in the dose-dependence of TBB-induced radiosensitization where the fibroblasts but not the tumor cells exhibited a significant threshold-type behaviour. Whether this difference is maintained when high radiation doses are delivered by small consecutive doses in a fractionation schedule is presently under investigation. In the absence of more pronounced differentially expressed phenotypes in tumor cells versus normal cells, however, a potential therapeutic benefit of a TBB-radiation combination can presently not be implied.

## Conclusion

The data imply a role of CK2 in γH2AX dephosporylation, most likely through its known ability to stimulate PP2A phosphatase, while DSB physical rejoining was unaffected. The slight but definite enhanced clonogenic radiation response by TBB does apparently not result from interference with an apoptosis suppression function of CK2 in these cells but could reflect inhibitor-induced uncoupling of DNA damage response decay from break ligation.

## Conflict of interests

The authors declare that they have no competing interests.

## Authors' contributions

FZ carried out the immunofluorescence experiments, the pulsed-field electrophoresis and the clonogenic assays and also drafted the manuscript. ME carried out the apoptosis measurements and helped by the gamma H2AX experiments. PH and JD participated importantly in the conception of the study and provided informatics and support with statistics for data analysis. KW conceived of the study, participated in its design and helped to draft the manuscript. All authors read and approved the final manuscript.
